# Rescuer Exertion and Fatigue Using Two-Thumb vs. Two-Finger Method During Simulated Neonatal Cardiopulmonary Resuscitation

**DOI:** 10.3389/fped.2020.00133

**Published:** 2020-04-02

**Authors:** Claire Reynolds, Jennifer Cox, Vicki Livingstone, Eugene Michael Dempsey

**Affiliations:** ^1^Neonatal Intensive Care Unit, Department of Paediatrics and Child Health, University College Cork, Cork, Ireland; ^2^INFANT, Irish Centre for Fetal and Neonatal Translational Research, University College Cork, Cork, Ireland

**Keywords:** newborn, fatigue, two finger technique, two thumb technique, CPR–cardiopulmonary resuscitation

## Abstract

**Background:** Rescuer fatigue during neonatal CPR can affect CPR quality leading to reduced cerebral and myocardial perfusion.

**Aim:** To investigate rescuer fatigue during simulated neonatal CPR using both objective (heart rate and cardiac output) and subjective measures.

**Methods:** A randomized crossover manikin study performed. Nineteen doctors working in neonatology were randomized to (a) two-thumb term, (b) two-finger term, (c) two-thumb preterm, or (d) two-finger preterm group. Cardiac output and heart rate were measured with a non-invasive cardiac output monitor. A Likert scale assessed participants' level of perceived exertion.

**Results:** In the preterm group, the mean change in HR from rest to 5 min in the TT group was 11.58 bpm (SD 6.22) vs. 9.94 bpm (SD 8.48), (*p*-value 0.36). There was no difference in change in CO, 2.10 (SD 1.15) in the TT group vs. 1.39 (SD 1.63) in TF group (*p* value 0.23). There was no difference in BORG RPE rating. In the term group, the mean change in HR from rest to 5 min was 15 bpm (SD 8.40) in TT group and 13 bpm (SD 7.86) in TF group, (*p*-value 0.416). The median change in CO from rest to 5 min was 1.50 (0.78 to 2.42 IQR) in TT group vs. 1.60 (0.65 to 3.0 IQR) in TF group.

**Conclusion:** Providing chest compressions is associated with an increase in both heart rate and cardiac output. We did not identify difference between objective and subjective measures of fatigue between either technique in a preterm or term model.

## Introduction

The majority of newborns transition successfully from intrauterine to extrauterine life without any assistance. Less than 1% of all newborns will require extensive neonatal resuscitation involving chest compressions (CC) and medication administration at birth (1). CC are indicated for those newborns whose heart rate (HR) remains less than 60 beats per minute despite adequate ventilation. The American Heart Association (AHA) identifies two different methods of delivering chest compressions; (a) the two-thumb (TT) method and (b) the two-finger (TF) method. The TT technique consists of thumbs placed together (side by side or one on top of the other), pointing cephalad, on the lower third of the sternum while the hands encircle the chest and fingers support the back. The TF methods consists of the tips of the middle and either ring or index finger placed on the lower third of the sternum perpendicular to the chest. The two-thumb method is favored by the AHA as it results in higher blood pressure and coronary perfusion pressures and can also be delivered from the head of the bed during umbilical catheter insertion (1). CC should be performed using a 3:1 compression to ventilation (C:V) ratio, with 90 CC and 30 inflations to achieve a total of 120 events/min (2).

The most important determinant of effective cardiopulmonary resuscitation (CPR) is quality of CC (3). High quality CC improve cerebral and myocardial perfusion and thus survival rate (4). Quality depends on (A) optimal compression: ventilation (C:V) ratio, (B) adequate CC rate, (C) depth of CC and (D) full recoil between compressions (4, 5), all of which are influenced by provider performance.

Provider fatigue impacts negatively on performance and CC quality (6–10). Fatigue is difficult to characterize and measure, and is often subjective. Fatigue has been defined as “extreme tiredness resulting from mental or physical exertion.” Recently, a number of simulation studies have investigated the impact of provider fatigue during CPR using objective assessments of changes in CC quality and subjective assessments of perceived fatigue measured by Likert scales. Additionally, physiological parameters/responses assessing provider exertion have also been used as an indirect measure or surrogate marker of fatigue.

CC duration, C:V method, and CC rate during CPR and the associated impact on provider fatigue have been extensively investigated to date. Sugaran et al. reported rescuer fatigue, evidenced by CC depth decay, after only 90 s of CPR, with no change in CC rate, during in-hospital CPR (10). Ashton et al. reported adverse effects on CC quality when performed without interruption over a 3 min period (9). Thus, the current pediatric resuscitation guidelines recommend providers rotate every 2 minutes during CC delivery in order to avoid significant rescuer fatigue and poor CC quality (9, 11, 12). No such recommendation exists in the neonatal resuscitation guidelines. Boldingh et al. compared the effect of 3:1 C:V CPR with CCaV (continuous CC with asynchronous ventilation) at 120 CC/min on rescuer fatigue during simulated neonatal CPR. In their study, physiological changes in HR/RR/MAP, perceived level of fatigue (likert scale) and manikin CC measures were employed and greater levels of rescuer fatigue during CCaV at 120 CC per min were reported (13). Li et al. assessed rescuer fatigue during (i) 3:1 C:V ratio, (ii) CCaV at 90 CC per min, and (iii) CCaV at 120 CC per min, using peak pressure and CC rate, however, unlike Boldingh et al., they found both 3:1 C:V and CCaV CPR to be equally fatiguing (14).

Different CC techniques (TT vs. TF method) can also impact on rescuer fatigue, however, to date limited research has investigated this. The TT technique is the favored technique during neonatal CPR; producing significantly higher systolic, diastolic, mean arterial, and pulse pressures than the TF technique (15). It also delivers higher quality compressions over a longer time period, with superior CC depth and less variability with each compression (3, 16, 17).

Thus, the primary aim of this study was to compare physiological measures of provider exertion, using two different CC techniques (TT vs. TF) during 5 min of simulated neonatal CPR on term and preterm manikins. Exertion was objectively measured using changes in particpants' HR and cardiac output (CO), which provided a surrogate marker of provider fatigue. A subjective assessment of provider fatigue using the Borg Scale of Perceived Exertion was also employed. We hypothesized that TF technique would exert our participants more, resulting in higher CO and HR levels over time, and higher levels of perceived fatigue, compared with the TT technique.

## Methods

### Study Design and Participants

The study was a randomized crossover manikin study conducted over a 1 month period in Cork University Maternity Hospital (CUMH) in 2018. The study protocol was approved by the Cork University's Research Ethics committee (approval number ECM 4 (ff) 07/03/2018). Consultants and junior doctors in training working in the neonatology department in CUMH were recruited for the study. All participants were required to have up-to-date Neonatal Resuscitation Programme (NRP) training and certification. Exclusion criteria included any underlying medical conditions contraindicating the exertion associated with CPR. Voluntary written informed consent was obtained from all participants. The study included 19 participants (Consultants *n* = 3, Junior Doctors *n* = 16).

### Study Protocol

The study was a cross over design. All participants performed four series of CC: (a) Two-thumb (TT) term, (b) Two-thumb (TT) preterm, (c) Two-finger (TF) term, (d) Two-finger (TF) preterm. Each participant was randomized to either the TT or TF technique. They would perform this technique on the term manikin first and then the preterm manikin, before crossing over and repeating this procedure using the alternative CC technique. A minimum period of 20 min was provided between each series of chest compressions, allowing participants to recover and HR/CO to return to baseline before commencing the next simulation. Participants each performed 5 min of simulated chest compressions at a 3:1 compression-to-ventilation ratio, aiming to achieve 90 CCs and 30 rescue breaths per minute (120 events/minute).

Prior to starting, participants were given standardized instructions about correct hand placement on lower third of sternum and chest compression depth of one-third the anterior posterior diameter. They were also familiarized with the BORG rate of perceived exertion scale and instructed to rate their level of perceived exertion at 1-min intervals throughout the simulation. CO and HR of the participants was measured with a non invasive CO monitor (Cheetah Starling NICOM). Four NICOM non-invasive sensor pads were applied to the participant's thorax prior to commencing the simulation which allowed for continuous monitoring of CO and HR throughout the simulation. HR and CO were recorded at baseline/rest and at minute intervals over the 5 min simulation.

We did not objectively assess the quality of the chest compressions, which would have provided an alternative method of assessment of fatigue. However, a single consistent observer (CR) supervised the simulation and gave verbal motivation and technique feedback to participants to optimize performance and ensure delivery of consistent quality compressions throughout.

### Data Collection/Measurements

Data was collected on participant demographics including age, gender, weight, height, and BMI. We evaluated objective levels of fatigue using NICOM sensors which collected data on CO and HR before initiation of CPR and then at minute intervals throughout the 5 min simulation. Qualitative data on perceived level of fatigue was evaluated at 1 min intervals using a validated likert scale [Borg rate of perceived exertion (RPE)]. The scale starts with “no feeling of exertion,” which rates a 6, and ends with “very, very hard,” which rates a 20. Moderate activities register 11 to 14 on the Borg scale (“fairly light” to “somewhat hard”), while vigorous activities usually rate a 15 or higher (“hard” to “very, very hard”). This scale is based on the high correlation between the scale and HR (i.e., multiplying the Borg score by 10, gives an approximate HR for a particular level of activity).

### Statistical Analysis

An a priori sample size calculation indicated that a sample of 24 participants was necessary to detect a medium-large effect size (Cohen's *f* = 0.60)^1^ in a paired t-test comparing relative change (%) in CO when using two thumb compression with relative change (%) in CO when using two finger compression with a power of 80%, a level of significance of 0.05 and a 2-tailed test.

Continuous data was described using mean and standard deviation (SD) when the data was normally distributed or the median and interquartile range (IQR) when the data was not normally distributed. For each group (term, preterm) separately, the paired *t*-test or Wilcoxon signed-rank test was used to compare change in CO and HR from rest to 5 min between the two techniques (TT, TF). Relationships between Borg (RPE) and both CO and HR at 5 min were measured using Spearman's correlation coefficient. All tests were two-sided and a *p*-value < 0.05 was considered statistically significant. All statistical analysis was performed using IBM SPSS Statistics (version 25.0, IBM Corp, Armonk, NY, U.S.A.).

## Results

There were 19 participants recruited for the study (females = 10). The median (IQR) age was 31 years (29.0 to 34.0). The median weight (IQR) was 67.1 kg (61.8 to 80.0) and the median BMI (IQR) was 24.6 (21.8 to 26.7).

Due to technical problems with the NICOM leads, we had to exclude some of the participants at 5-min CO/HR levels due to potential artifact and this is represented in Tables 1–**6**.

**Table 1:** Preterm Model: HR, CO, and BORG Scores for each CC technique.

	**TT**	**TF**
Mean HR bpm rest (SD)	80.00 (9.97); *n* = 19	82.53 (11.16); *n* = 19
Mean HR bpm 5 min (SD)	92.66 (12.90); *n* = 17	91.53 (12.87); *n* = 15
Mean CO l/min rest (SD)	6.68 (1.54); *n* = 19	7.12 (1.85); *n* = 19
Mean CO l/min 5 min (SD)	8.59 (2.23); *n* = 17	8.57 (1.44); *n* = 15
Mean max CO l/min (SD)	8.50 (2.11); *n* = 19	8.43 (1.60); *n* = 19
Mean Borg 5 min (SD)	11.11 (2.30); *n* = 19	11.79 (2.32); *n* = 19

### Preterm Model

The mean resting HR and CO were similar for both CC techniques (Table 1). The mean change in HR from rest to 5 min was higher for the TT technique (11.58 pm (SD 6.22) for TT technique vs. 9.94 bpm (SD 8.48) for TF technique). However the difference was not statistically significant (*p* = 0.365, Table 2). The mean change in CO from rest to 5 min was also higher for the TT technique (2.10l/min (SD 1.15) for TT technique vs. 1.39l/min (SD 1.63) for TF technique). However the difference was not statistically significant (*p* = 0.236, Table 2).

**Table 2:** Preterm model: changes in HR and CO from rest to 5 min.

	***n***	**TT change 5 min-rest Mean (SD)**	**TF change 5 min-rest Mean (SD)**	**Difference in means (95%CI)**	***p*-value**
HR bpm	17	11.58 (6.22)	9.94 (8.48)	1.65 (−2.10 to 5.39)	0.365
CO l/min	14	2.10 (1.15)	1.39 (1.63)	0.71 (−0.53 to 1.96)	0.236

Max CO levels reached in both groups were similar (Table 1). Mean 5 min Borg rating was similar for both CC techniques; each having an average score of 11 which correlates with “fairly light” (Table 1). There was a weak, positive relationship between Borg RPE scale and CO for both techniques. There was also a weak, positive relationship between Borg RPE and HR for the TT technique. No relationship was found between Borg RPE and HR for the TF technique (Table 3).

**Table 3:** Preterm model: Borg scores and relationship to CO and HR.

		***N***	**Spearman's correlation coefficient**	***p*-value**
Borg 5 min (TT)	HR 5 min (TT)	18	0.259	0.299
Borg 5 min (TF)	HR 5 min(TF)	18	−0.044	0.862
Borg 5 min (TT)	CO 5 min (TT)	17	0.145	0.578
Borg 5 min (TF)	CO 5 min (TF)	15	0.187	0.503

### Term Model

Similar to the preterm group, resting HR and CO were similar between the two techniques (Table 4). The mean change in HR from rest to 5 min was higher for the TT technique (15.30 bpm (SD 8.40) for TT technique vs. 13.20 bpm (SD 7.86) for TF technique). However the difference was not statistically significant (*p* = 0.416, Table 5). The median change in CO from rest to 5 min was similar for both techniques (1.50 l/min (IQR: 0.78 to 2.42) for TT technique vs. 1.60 l/min (IQR: 0.65 to 3.0) for TF technique, *p* = 0.442, Table 5).

**Table 4:** Term model.

	**TT**	**TF**
Mean HR bpm rest (SD)	84.11 (11.87); *n* = 19	81.84 (9.57); *n* = 19
Mean HR bpm 5 min (SD)	99.50 (13.0); *n* = 16	94.18 (8.91); *n* = 17
Mean CO l/min rest (SD)	6.78 (1.46); *n* = 19	6.91 (1.66); *n* = 19
Mean CO l/min 5 min (SD)	8.46 (2.20); *n* = 16	9.19 (1.97); *n* = 17
Mean Max CO l/min (SD)	8.67 (2.11); *n* = 19	9.06 (2.01); *n* = 19
BORG 5 min mean (SD)	13.90 (2.98); *n* = 19	15.21 (3.16); *n* = 19

*HR, CO and BORG Scores for each CC technique*.

**Table 5:** Term model: changes in HR and CO from rest to 5 min.

	***N***	**TT change 5 min-rest Mean (SD)**	**TF change 5min-rest Mean (SD)**	**Difference in means (95%CI)**	***p*-value**
HR bpm	17	15.30 (8.40)	13.20 (7.86)	2.18 (-3.35 to 7.70)	0.416
	***N***	**TT change 5 min-rest Median (IQR)**	**TF change 5min-rest Median (IQR)**		***p*****-value**
CO l/min	16	1.50 (0.78 to 2.42)	1.60 (0.65 to 3.0)		0.442

Max CO levels reached in both groups were similar (Table 4). Borg at 5 min was higher for the TF technique compared to the TT technique and was rated as 15 (hard) compared to 14 (somewhat hard), Table 4. There was a strong, positive correlation between Borg RPE at 5 min and HR for the TF technique (r_s_ = 0.536, *p* = 0.022), Table 6. This relationship was not evident for the TT technique (r_s_ = 0.159, *p* = 0.528). There was a weak, positive relationship between Borg RPE scale and CO for the TT techniques while there was a weak, negative relationship for the TF technique.

**Table 6:** Term Model: BORG scores and relationship to CO and HR.

		***n***	**Spearman's correlation coefficient (rs)**	***p*-value**
Borg 5 min (TT)	HR 5 min (TT)	18	0.159	0.528
Borg 5 min (TF)	HR 5 min (TF)	18	0.536	0.022
Borg 5 min (TT)	CO 5 min (TT)	16	0.145	0.592
Borg 5 min (TF)	CO 5 min (TF)	17	−0.258	0.318

## Discussion

In this randomized crossover trial of physicians performing simulated CC, we found no difference in objective measures of exertion in participants performing chest compressions with either the two thumb or two finger technique. There were no differences in HR change, CO change, max CO achieved between the two techniques, in both a preterm and term manikin model. We also found no difference in BORG scales as a subjective measure of exertion/fatigue.

Very few studies have compared rescuer fatigue/exertion using two different compression techniques. Douvanas et al. systematically reviewed all available studies conducted between 2010 and 2015 comparing TT and TF technique on quality of CCs, ventilation, and rescuer fatigue. Four studies in the review compared rescuer fatigue between TT and TF technique. Different methods including physiological responses, perceived fatigue using likert scales, and changes in CC quality were employed to assess provider fatigue (3). They concluded that the majority of studies reviewed, favored the TT technique over the TF technique for infant and neonatal resuscitation, because it provides higher quality CC over a longer period, achieves less variability in hand placement and reduces rescuer fatigue (3).

Huynh et al. compared the TT and TF technique on an elevated surface or on the floor (18). A subjective self-assessment of preference and fatigue found that more subjects preferred the TT (60%) over the TF (40%) technique because of increased stability and ability to control the depth of compressions, easier placement of hands during CC, and less fatiguing. Similarly, Smereka et al. performed a randomized crossover manikin trial comparing TT and TF technique on CC quality during 2 min of CPR. Using a 100-degree scale (1 = no fatigue; 100 = extreme fatigue), TF technique achieved 72 points (IQR 61–77) vs. 47 points (IQR 40–63) for TT (*p* = 0.034) and was thus deemed to be more fatiguing (16).

In our current study, we employed Borg rating of perceived exertion (RPE) to subjectively assess provider fatigue. Borg's RPE is a widely used psychophysical tool which assesses subjective perception of effort/fatigue during exercise and is considered an affordable and valid tool for monitoring and prescribing exercise regardless of age, gender, type of exercise and physical activity level (19). In both term and preterm groups, we found no significant difference in subjective measure of fatigue using either the TT or TF CC technique. The preterm 5-min Borg score was 11 in both CC groups which correlates to “fairly light.” In the term group, the Borg score was rated slightly higher during the TF technique compared with the TT technique (15 or “hard” vs. 14 or “somewhat hard”). The difference in Borg ratings between the term and preterm model is likely related to the difference in size of each manikin; the term manikin is larger and requires greater force to deliver quality CC.

We also compared Borg RPE scores and CO, in both term and preterm groups, and reported a weak correlation between both regardless of CC technique employed. Assessing HR, we reported a weak positive correlation between 5 min Borg RPE and HR using the TT technique in both term and preterm groups. There was a strong correlation between 5 min Borg RPE and HR using the TF technique in the term group. It is interesting that Borg rating was not well correlated with HR given that it is considered a reliable tool to accurately capture participant HR level. Our participants were educated prior to the simulation to ensure appropriate understanding and use of the score. The lack of correlation reported could possibly be related to participants' perceived difficulty of performing the CC technique; some participants complained of cramping in their hands during both techniques as the simulation reached the 5 min mark and this could have been reflected in the BORG score rather than their level of true exertion as measured by HR and CO.

Changes in HR and CO, were used to assess exertion in participants throughout the simulation. In the preterm group there was no statistically significant mean change in HR or CO between the TT or TF techniques. Similarly in the term group, the mean change in HR between both CC techniques was not statistically significant. The median change in CO between both techniques also did not reach statistical significance. These findings need to be interpreted cautiously considering the number of participants enrolled and given that some data was excluded due to technical difficulties. We believe it is best interpreted as that objectives measures of exertion were no different between the two techniques with either manikin. Increasing exertion will ultimately result in reduced performance, and the fact that there was no difference one might assume that fatigue should then be no different between both techniques. It is difficult to draw any inferences from the difference between HR and CO between the two manikin types, other than to say that provider HR increase was greater in the term model, but provider CO was greater in the preterm model.

Jo et al. compared over-the-head two thumb (OTTT) encircling technique to the two-finger (TF). Their study compared CPR quality between the two CC techniques as well as comparing fatigue and perceived difficulty utilizing changes in RR, HR and a five-point Likert-scale-based survey (0 = very easy and 5 = very hard). They reported no significant change in HR or RR, however the fatigue score was lower after OTTT compared with the TF (20). In another study, Udassia et al. compared TT technique to TF technique during lone rescuer infant manikin CPR and found that the two thumb provided more effective compression depth and compression pressure compared to two-finger technique (21). They also evaluated rescuer fatigue by assessing increases in HR and RR following 2 min of CPR. A likert-scale based study questionnaire assessed for CC difficulty, ease of changing from compressions to ventilation and whether fatigue reduced CPR performance. (22) Similar to Jo et al., there were no significant differences in the average increase in HR or respiratory rate over 2 min of CPR between the two techniques. However, participants perceived that they achieved better compression rate and depth with the TT compared to the TF method (*p* < 0.05).

One of our study's strengths is that we used both subjective and objective measures to assess rescuer exertion/fatigue. Our subjective assessment was performed using Borg's RPE scale which is a valid tool used to assess subjective perception of effort/fatigue. Physiological measures including HR and CO were also included to assess provider exertion, an indirect measure of provider fatigue. To date, very few studies have assessed the difference in physiological response to different CC techniques. Two previous studies assessed HR and RR but none have assessed CO. Our inclusion of both term and preterm models in this study is also novel. All previous simulated CPR studies to date have been performed on term neonate or infant manikins.

There were a number of limitations in our study. Firstly, our sample size was small. We had anticipated a higher recruitment rate but this proved difficult. Also, due to the exclusion criteria, a number of our junior doctors in training were not eligible to take part in the study.

There were some technical issues with our NICOM leads during the simulation which resulted in missing data. This was possibly due to movement of simulating the CPR technique. Ultimately this resulted in some missing data which we were not able to retrieve once the CPR simulation was finished. Our biggest limitation, is that we did not formerly assess for quality of CPR delivered, which may have been compromised with onset of fatigue but we did have a sole person dedicated to assessing depth and quality of CC, and also gave verbal cues throughout the simulation to ensure quality was maintained. However we recognize that this limits our ability to quantify fatigue based on performance of either method. Finally, the study took place in a controlled simulated environment, and one could argue that the results could possibly be different in more stressful conditions.

Previous studies have advocated for CPR providers to rotate every 2–3 min in order to avoid fatigue. Providing CC is physically demanding, associated with increases in HR and CO above baseline, as well as provider exertion. However alternating methods may impact upon performance and this would need to be formally evaluated.

In conclusion, providing CC is physically demanding and is associated with an increase in both HR and CO from baseline levels. We did not identify any discernible difference between objective and subjective measures of fatigue between both techniques, either in a preterm or term model of CC. An awareness that there is a physical demand when performing CC is essential, and that alternating the role between providers during resuscitation may assist in avoiding rescuer exertion, regardless of the initial method employed. However, further studies are required to address this aspect of resuscitation.

## Data Availability Statement

The datasets generated for this study are available on request to the corresponding author.

## Ethics Statement

The study protocol was approved by the Cork University's Research Ethics committee (approval number ECM 4 (ff) 07/03/2018). Voluntary written informed consent was obtained from all participants.

## Author Contributions

All authors have made substantial contributions to the conception and design of the study, or acquisition of data, or analysis and interpretation of data, drafting the article or revising it critically, and final approval of the version to be submitted.

### Conflict of Interest

The authors declare that the research was conducted in the absence of any commercial or financial relationships that could be construed as a potential conflict of interest.
